# Supplementary material for: Inheritance of Some Traits in Crosses between Hybrid Tea Roses and Old Garden Roses
Source: Plants (Basel). 2024 Jun 28;13(13):1797. doi: 10.3390/plants13131797 (PMC11244027; doi:10.3390/plants13131797)
Supplement: Supplementary file 1 [file plants-13-01797-s001.zip › plants-3018815-supplementary.pdf]

plants

Supplementary Materials

Inheritance of some Traits in Crosses between Hybrid Tea Roses and Old Garden Roses

1.1. Supplementary Tables

Table S1. Heterosis and heterobeltiosis (%) of F_1 progenies for various traits.

F_1	Flower Stem Length		Petal Number		Flower Diameter		Bud Length	
Progeny	%Ht	%Hbt	%Ht	%Hbt	%Ht	%Hbt	%Ht	%Hbt
LxBR-1	-9.71	38.20	-17.65	-8.20	-32.02	-23.97	-34.52	-20.54
LxBR-2	-84.00	-13.80	-56.85	-51.90	-47.78	-41.60	-60.22	-51.73
LxBR-3	-47.86	11.50	-24.50	-15.84	-4.83	6.45	-19.83	-2.72
LxBR-4	-55.43	6.20	6.85	19.11	-31.03	-22.87	-36.24	-22.63
LxBR-5	-35.29	20.30	-21.56	-12.56	-32.02	-23.97	-42.30	-29.98
LxBR-6	-36.71	19.30	-45.59	-39.34	-33.50	-25.62	-41.59	-29.12
LxBR-7	-39.71	17.20	-57.65	-52.79	-43.05	-36.31	-56.43	-47.12
LxBR-8	-88.14	-16.70	0.00	11.48	-45.81	-39.39	-59.33	-50.65
LxBR-10	-50.57	9.60	-5.88	4.92	-22.17	-12.95	-29.52	-14.47
LxBR-11	-51.57	8.90	8.82	21.31	-6.40	4.68	-24.31	-8.16
LxBR-12	-86.43	-15.50	-31.18	-23.28	-49.46	-43.47	-58.20	-49.28
LxBR-13	-81.00	-11.70	26.47	40.98	-45.02	-38.51	-60.17	-51.66
LxBR-14	-49.29	10.50	-52.94	-47.54	-29.56	-21.21	-46.78	-35.42
LxBR-15	-52.57	8.20	-18.15	-8.75	-14.78	-4.68	-23.94	-7.71
LxBR-16	-44.71	13.70	-47.06	-40.98	-43.65	-36.97	-50.72	-40.20
LxBR-17	-43.71	14.40	0.00	11.48	-38.82	-31.57	-44.17	-32.25
LxBR-18	-30.14	23.90	-32.06	-24.26	-52.41	-46.78	-64.00	-56.31
LxBR-19	-31.57	22.90	-27.44	-19.11	10.05	23.09	10.57	34.18
LxBR-20	-33.14	21.80	5.88	18.03	-41.67	-34.77	-46.26	-34.79
LxBR-22	-30.43	23.70	-39.71	-32.79	-30.54	-22.31	-38.15	-24.94
LxBR-23	-39.14	17.60	-7.35	3.28	-43.25	-36.53	-50.57	-40.02
LxBR-24	-37.71	18.60	-14.71	-4.92	-28.28	-19.78	-35.83	-22.13
LxBR-25	-30.14	23.90	-0.97	10.39	-8.28	2.59	-23.93	-7.69
LxBR-26	-49.86	10.10	10.29	22.95	-35.17	-27.49	-39.09	-26.09
LxBR-28	-85.43	-14.80	-7.35	3.28	-57.54	-52.51	-66.28	-59.08
LxBR-30	-75.14	-7.60	-62.74	-58.46	-22.66	-13.50	-44.39	-32.52
LxBR-31	-49.86	10.10	-60.18	-55.61	-18.82	-9.20	-31.09	-16.38
LxBR-32	-48.43	11.10	-27.44	-19.11	-14.09	-3.91	-40.00	-27.19
LxBR-33	-45.43	13.20	-55.88	-50.82	-21.77	-12.51	-38.43	-25.28
LxBR-34	-66.43	-1.50	-15.68	-6.00	-50.34	-44.46	-62.70	-54.74
LxBR-35	-60.86	2.40	5.88	18.03	-51.63	-45.90	-63.81	-56.09
LxBR-36	-15.86	33.90	-31.47	-23.61	-21.28	-11.96	-26.81	-11.19

**LxBR-37**	-46.29	12.60	-30.88	-22.95	-24.83	-15.92	-37.85	-24.58
**LxBR-38**	-33.14	21.80	-53.91	-48.62	-31.53	-23.42	-42.11	-29.75
**LxBR-41**	-32.86	22.00	-53.91	-48.62	-42.17	-35.32	-59.15	-50.43
**LxBR-42**	-48.29	11.20	-58.82	-54.10	-10.15	0.50	-19.93	-2.83
**LxBR-43**	-55.86	5.90	-15.29	-5.57	-21.87	-12.62	-34.22	-20.18
**LxBR-44**	-32.71	22.10	2.94	14.75	-35.57	-27.93	-39.50	-26.58
**LxBR-45**	-36.57	19.40	39.71	55.74	-31.92	-23.86	-28.13	-12.79
**LxBR-46**	-38.29	18.20	81.38	102.20	-15.37	-5.34	-25.33	-9.39
**LxBR-48**	-43.43	14.60	13.74	26.79	-42.17	-35.32	-52.93	-42.88
**LxBR-49**	-47.86	11.50	5.88	18.03	-38.92	-31.68	-29.04	-13.89
**LxBR-50**	-44.71	13.70	-22.44	-13.54	1.48	13.50	-6.13	13.91
**LxBR-51**	-43.71	14.40	-11.76	-1.64	-14.19	-4.02	-31.69	-17.10
**LxBR-52**	-39.57	17.30	52.94	70.49	-32.22	-24.19	-37.30	-23.91
**LxBR-53**	-73.43	-6.40	-7.85	2.72	-51.53	-45.79	-59.00	-50.25
**LxBR-54**	-43.57	14.50	32.35	47.54	-45.81	-39.39	-57.04	-47.87
**LxBR-55**	-46.00	12.80	-43.38	-36.89	-17.93	-8.21	-33.57	-19.39
**LxBR-56**	-47.71	11.60	12.74	25.67	-11.43	-0.94	-30.72	-15.93
**LxBR-57**	-69.43	-3.60	221.56	258.46	-20.10	-10.63	-18.93	-1.62
**LxBR-58**	-44.57	13.80	-13.74	-3.84	-23.84	-14.82	-42.13	-29.78
**LxBR-59**	-65.00	-0.50	108.82	132.79	-46.50	-40.17	-58.26	-49.35
**LxBR-61**	-45.43	13.20	54.91	72.69	-25.52	-16.69	-29.89	-14.92
**LxBR-62**	-49.14	10.60	14.71	27.87	-27.09	-18.46	-27.89	-12.49
**LxBR-63**	-35.57	20.10	45.09	61.74	-23.25	-14.16	-29.93	-14.97
**LxBR-64**	-46.43	12.50	-12.74	-2.72	-27.29	-18.68	-23.24	-6.85
**LxBR-65**	-49.86	10.10	-39.21	-32.23	-25.12	-16.25	-33.69	-19.53
**LxBR-66**	-41.29	16.10	-48.03	-42.07	-23.45	-14.38	-41.74	-29.30
**LxBR-67**	-31.43	23.00	-30.59	-22.62	-8.67	2.15	-25.59	-9.71
**LxBR-68**	-32.71	22.10	10.79	23.51	-19.80	-10.30	-34.35	-20.34
**LxBR-70**	-32.29	22.40	-20.59	-11.48	-41.28	-34.33	-50.41	-39.82
**LxBR-71**	-51.29	9.10	-1.97	9.28	-47.78	-41.60	-54.96	-45.35
**LxBR-72**	-44.43	13.90	-28.44	-20.23	-51.92	-46.23	-51.91	-41.64
**LxBR-73**	-40.71	16.50	-28.44	-20.23	-19.21	-9.64	-34.80	-20.88
**LxBR-74**	-55.43	6.20	67.65	86.89	-6.80	4.24	-25.09	-9.10
**LxBR-75**	-86.43	-15.50	-50.00	-44.26	-45.81	-39.39	-57.13	-47.98
**LxBR-76**	-50.71	9.50	38.24	54.10	-12.41	-2.04	-26.31	-10.58
**LxBR-78**	-52.29	8.40	17.15	30.59	-13.89	-3.69	-32.00	-17.48
**LxBR-79**	-46.00	12.80	-25.00	-16.39	-21.87	-12.62	-37.76	-24.47
**LxBR-80**	-42.71	15.10	45.09	61.74	-5.71	5.45	-31.83	-17.28
**LxBR-81**	-41.57	15.90	56.85	74.85	-36.26	-28.71	-46.67	-35.28
**LxBR-82**	-43.71	14.40	-13.74	-3.84	-31.72	-23.64	-45.22	-33.53
**LxBR-83**	-35.57	20.10	-5.88	4.92	-29.56	-21.21	-47.39	-36.16
**LxBR-84**	-34.00	21.20	-49.26	-43.44	-48.18	-42.04	-56.78	-47.55

**LxBR-85**	-31.71	22.80	-26.47	-18.03	-35.57	-27.93	-51.48	-41.12
**LxBR-86**	-36.00	19.80	43.15	59.57	-62.76	-58.35	-38.81	-25.75
**LxBR-87**	-80.57	-11.40	-50.97	-45.34	-59.21	-54.38	-72.63	-66.79
**LxBR-88**	-48.43	11.10	-11.76	-1.64	-45.02	-38.51	-49.11	-38.25
**LxCR-1**	-49.29	10.50	-60.82	-41.98	-31.24	-26.51	-30.06	-23.70
**LxCR-2**	-45.00	13.50	12.03	65.91	-16.48	-10.73	-23.72	-16.79
**LxCR-3**	-35.43	20.20	-58.76	-38.93	-26.52	-21.47	-21.17	-14.00
**LxCR-5**	-2.71	43.10	-60.82	-41.98	-14.25	-8.35	-20.91	-13.72
**LxCR-9**	12.86	54.00	-65.98	-49.62	-37.68	-33.39	-36.22	-30.42
**LxCR-10**	-51.29	9.10	-86.60	-80.15	-35.88	-31.47	-40.41	-34.99
**SxBR-1**	-54.71	13.50	66.67	17.45	-47.26	-35.88	-42.67	-28.49
**SxBR-2**	-62.94	6.50	-79.79	-85.76	-39.84	-26.86	-39.41	-24.43
**SxBR-4**	-43.41	23.10	9.09	-23.13	-34.68	-20.59	-37.09	-21.53
**SxBR-5**	-44.12	22.50	-61.61	-72.94	-54.52	-44.71	-48.88	-36.24
**SxBR-6**	-45.65	21.20	-9.09	-35.94	-44.76	-32.84	-42.33	-28.06
**AxBR-1**	-32.00	19.20	-20.43	-18.38	16.72	-5.10	-15.08	6.60
**AxBR-2**	-33.69	18.10	55.83	59.82	4.18	-15.29	-27.05	-8.43
**AxBR-3**	3.54	42.30	-28.13	-26.28	-3.68	-21.68	-30.37	-12.60
**AxBR-4**	-70.15	-5.60	-52.50	-51.28	-31.64	-44.42	-51.19	-38.72
**AxBR-5**	-49.85	7.60	-62.50	-61.54	6.07	-13.75	-22.10	-2.21
**AxBR-6**	-8.77	34.30	45.00	48.72	-34.03	-46.36	-46.69	-33.09
**AxBR-7**	-39.69	14.20	53.00	56.92	-40.10	-51.29	-49.93	-37.15
**AxBR-8**	-45.38	10.50	-44.18	-42.74	-24.38	-38.51	-37.19	-21.15
**AxBR-9**	-44.46	11.10	-34.78	-33.10	10.85	-9.87	-11.31	11.34
**AxBR-11**	-70.92	-6.10	-21.88	-19.87	-49.75	-59.14	-62.47	-52.89
**AxBR-12**	-48.77	8.30	34.18	37.62	-9.65	-26.54	-27.12	-8.51
**AxCR-4**	-45.23	10.60	108.33	12.61	-48.58	-44.79	-59.49	-54.04
**AxCR-5**	-60.62	0.60	-19.38	-56.42	-22.83	-17.14	-33.29	-24.31
**AxCR-6**	-45.85	10.20	-36.25	-65.54	-0.34	7.00	-9.14	3.10
**AxCR-9**	-40.46	13.70	-15.00	-54.05	-32.53	-27.56	-48.73	-41.83
**SAxBR-1**	-44.77	10.90	-50.00	-34.62	-48.07	-37.89	-48.03	-32.81
**SAxBR-2**	-41.23	13.20	-27.45	-5.13	-48.74	-38.69	-54.81	-41.58
**SAxBR-3**	-35.23	17.10	-39.86	-21.36	-32.35	-19.10	-43.20	-26.57
**SAxBR-4**	-30.31	20.30	-30.73	-9.41	-32.94	-19.80	-47.56	-32.20
**SAxBR-5**	-5.85	36.20	-33.98	-13.67	-40.92	-29.35	-46.70	-31.09
**SAxBR-7**	-26.31	22.90	-79.22	-72.82	-31.76	-18.39	-44.67	-28.46
**SAxBR-8**	-30.31	20.30	33.33	74.36	-34.62	-21.81	-49.94	-35.27
**SAxBR-9**	-2.00	38.70	-51.76	-36.92	-38.07	-25.93	-48.42	-33.31
**SAxBR-10**	2.92	41.90	-22.22	1.72	-0.92	18.49	-15.58	9.15
**SAxBR-11**	-81.54	-13.00	-50.98	-35.90	-60.84	-53.17	-66.31	-56.44
**SAxBR-12**	-50.77	7.00	-70.39	-61.28	-2.27	16.88	-20.06	3.35
**SAxBR-13**	-26.92	22.50	-68.29	-58.54	-27.48	-13.27	-43.11	-26.44

**SAxBR-14**	4.77	43.10	30.73	70.95	-24.79	-10.05	-42.20	-25.27
**MxBR-1**	-58.33	12.50	-8.32	-6.65	-42.95	-35.24	-55.00	-44.24
**MxBR-2**	-65.33	6.20	-46.43	-45.45	-21.14	-10.49	-32.00	-15.74
**MxBR-3**	-56.22	14.40	0.00	1.82	-31.62	-22.38	-49.98	-38.02
**MxBR-4**	-54.33	16.10	43.57	46.18	-10.38	1.73	-24.16	-6.02
**MxBR-6**	-46.78	22.90	29.46	31.82	-28.67	-19.03	-31.33	-14.91
**MxBR-7**	-49.67	20.30	-7.14	-5.45	-44.00	-36.43	-61.77	-52.63
**MxBR-8**	-57.00	13.70	23.82	26.07	-11.33	0.65	-28.19	-11.02
**MxBR-9**	-65.33	6.20	73.82	76.98	-52.10	-45.62	-47.65	-35.13
**MxBR-10**	-61.56	9.60	28.57	30.91	-43.05	-35.35	-50.05	-38.11
**MxBR-11**	-38.22	30.60	-74.61	-74.15	-38.76	-30.49	-40.95	-26.83
**MxBR-12**	-56.67	14.00	48.82	51.53	-45.14	-37.73	-64.26	-55.72
**MxBR-13**	-54.44	16.00	-25.00	-23.64	-25.90	-15.89	-37.81	-22.93
**MxBR-14**	-58.33	12.50	-11.43	-9.82	-7.24	5.30	-20.00	-0.87
**MxBR-15**	-49.44	20.50	21.43	23.64	-38.76	-30.49	-47.58	-35.04
**MxBR-16**	-49.89	20.10	-36.43	-35.27	-17.14	-5.95	-33.25	-17.28
**MxBR-17**	-46.56	23.10	1.43	3.27	-24.00	-13.73	-38.18	-23.39
**MxBR-18**	-3.11	62.20	23.82	26.07	-16.10	-4.76	-30.74	-14.17
**MxBR-19**	-79.78	-6.80	-21.43	-20.00	-34.95	-26.16	-45.72	-32.74
**MxBR-20**	-65.89	5.70	-26.79	-25.45	-40.86	-32.86	-46.81	-34.09
**MxBR-21**	-70.22	1.80	-8.32	-6.65	-53.24	-46.92	-64.65	-56.20
**MxBR-22**	-75.22	-2.70	53.57	56.36	-25.05	-14.92	-43.56	-30.07
**MxBR-23**	-60.89	10.20	107.14	110.91	-21.43	-10.81	-52.46	-41.09
**MxBR-24**	-57.33	13.40	8.32	10.29	-48.86	-41.95	-57.58	-47.43
**MxBR-25**	-59.22	11.70	-7.14	-5.45	-44.00	-36.43	-55.35	-44.67
**MxBR-26**	-56.44	14.20	79.75	83.02	-33.90	-24.97	-47.98	-35.54
**MxBR-27**	-46.44	23.20	-54.29	-53.45	-24.10	-13.84	-46.49	-33.70
**MxBR-28**	-48.67	21.20	-35.71	-34.55	-49.05	-42.16	-48.18	-35.78
**MxBR-29**	-50.22	19.80	81.25	84.55	-43.43	-35.78	-55.70	-45.11
**MxBR-30**	-48.67	21.20	228.57	234.55	-22.57	-12.11	-34.98	-19.43
**MxBR-31**	-26.11	41.50	3.57	5.45	-23.05	-12.65	-37.30	-22.30
**MxBR-32**	-56.44	14.20	-60.71	-60.00	-42.95	-35.24	-51.77	-40.24
**MxBR-33**	-63.22	8.10	-15.46	-13.93	-27.81	-18.05	-38.81	-24.17
**MxBR-34**	-60.33	10.70	120.25	124.25	-0.29	13.19	-14.65	5.76
**MxBR-35**	-79.78	-6.80	-28.57	-27.27	-36.29	-27.68	-53.93	-42.91
**MxBR-36**	-59.78	11.20	-3.57	-1.82	-30.19	-20.76	-33.32	-17.37
**MxBR-37**	-55.56	15.00	17.86	20.00	-49.33	-42.49	-57.35	-47.15
**MxBR-38**	-59.44	11.50	-53.57	-52.73	-20.76	-10.05	-36.12	-20.85
**MxBR-39**	-49.89	20.10	18.57	20.73	-19.81	-8.97	-20.12	-1.02
**MxBR-40**	-46.67	23.00	27.68	30.00	-29.14	-19.57	-51.86	-40.35
**MxBR-41**	-66.00	5.60	-23.21	-21.82	-9.81	2.38	-22.35	-3.78
**MxBR-43**	-49.33	20.60	82.86	86.18	-45.52	-38.16	-64.07	-55.48

**MxBR-44**	-32.33	35.90	128.57	132.73	-29.43	-19.89	-40.61	-26.41
**MxBR-45**	-56.00	14.60	-7.14	-5.45	-17.81	-6.70	-26.86	-9.37
**MxBR-46**	-58.33	12.50	134.29	138.55	-6.48	6.16	-21.40	-2.61
**MxBR-47**	-27.56	40.20	46.43	49.09	-24.10	-13.84	-34.47	-18.80
**MxBR-48**	-59.11	11.80	73.82	76.98	-15.33	-3.89	-32.95	-16.91
**MxBR-49**	-52.78	17.50	-28.57	-27.27	-27.90	-18.16	-36.00	-20.70
**MxBR-50**	-49.11	20.80	152.68	157.27	-17.24	-6.05	-43.77	-30.33
**MxBR-51**	-47.67	22.10	-43.75	-42.73	-32.95	-23.89	-45.67	-32.67
**MxBR-52**	-58.22	12.60	81.43	84.73	-41.62	-33.73	-47.74	-35.24
**MxBR-53**	-53.44	16.90	13.11	15.16	-13.62	-1.95	-27.37	-10.00
**MxBR-54**	-25.22	42.30	-65.36	-64.73	-45.05	-37.62	-41.04	-26.93
**MxBR-56**	-52.44	17.80	105.96	109.71	-7.90	4.54	-26.86	-9.37
**MxBR-57**	-59.78	11.20	0.00	1.82	-9.14	3.14	-27.37	-10.00
**MxBR-58**	-56.11	14.50	2.68	4.55	-24.38	-14.16	-31.14	-14.67
**MxBR-59**	-55.44	15.10	0.00	1.82	-45.33	-37.95	-47.54	-35.00
**MxBR-60**	-70.11	1.90	-10.71	-9.09	-48.76	-41.84	-57.72	-47.61
**MxBR-61**	-60.44	10.60	5.96	7.89	-44.86	-37.41	-46.82	-34.11
**MxBR-63**	-58.00	12.80	10.71	12.73	-25.43	-15.35	-35.12	-19.61
**MxBR-64**	-71.89	0.30	-71.43	-70.91	-61.14	-55.89	-62.81	-53.91
**MxBR-65**	-61.56	9.60	-3.57	-1.82	-51.33	-44.76	-59.75	-50.13
**MxBR-66**	-50.89	19.20	50.00	52.73	-46.67	-39.46	-49.63	-37.59
**MxBR-67**	-54.67	15.80	-26.43	-25.09	-38.10	-29.73	-49.72	-37.70
**MxBR-68**	-56.67	14.00	-19.04	-17.56	-26.95	-17.08	-35.65	-20.26
**MxBR-69**	-54.22	16.20	120.21	124.22	-37.62	-29.19	-42.79	-29.11
**MxBR-70**	-49.22	20.70	-58.04	-57.27	-47.24	-40.11	-49.98	-38.02
**MxBR-71**	-52.67	17.60	-45.25	-44.25	-31.24	-21.95	-35.98	-20.67
**MxBR-72**	-46.89	22.80	-8.32	-6.65	-29.62	-20.11	-38.42	-23.70
**MxBR-73**	-87.56	-13.80	-3.57	-1.82	-56.29	-50.38	-66.35	-58.30
**MxBR-74**	-27.22	40.50	-53.57	-52.73	-18.76	-7.78	-34.82	-19.24
**MxBR-75**	-52.22	18.00	3.57	5.45	-50.29	-43.57	-44.72	-31.50
**MxBR-77**	-50.89	19.20	-47.14	-46.18	-7.14	5.41	-18.89	0.50
**MxBR-78**	-93.00	-18.70	28.57	30.91	-49.14	-42.27	-62.63	-53.70
**MxBR-79**	-60.56	10.50	79.75	83.02	-50.76	-44.11	-64.19	-55.63
**MxBR-80**	-64.56	6.90	-50.00	-49.09	5.71	20.00	-15.21	5.07
**MxBR-81**	-57.00	13.70	-42.86	-41.82	-25.05	-14.92	-37.21	-22.20
**MxBR-82**	-59.78	11.20	7.14	9.09	-1.71	11.57	-28.33	-11.20
**MxBR-83**	-63.11	8.20	-28.57	-27.27	2.29	16.11	-6.40	15.98
**MxBR-84**	-54.22	16.20	-34.71	-33.53	-18.48	-7.46	-38.63	-23.96
**MxBR-85**	-49.33	20.60	39.29	41.82	-51.52	-44.97	-45.14	-32.02
**MxBR-87**	-31.89	36.30	75.00	78.18	-20.48	-9.73	-28.05	-10.85
**MxBR-88**	-60.44	10.60	-17.86	-16.36	-27.62	-17.84	-42.53	-28.78
**MxBR-89**	-62.67	8.60	3.57	5.45	-32.86	-23.78	-42.37	-28.59

**MxBR-90**	-58.33	12.50	25.00	27.27	-22.67	-12.22	-30.68	-14.11
**MxBR-91**	-57.22	13.50	123.82	127.89	-45.05	-37.62	-55.35	-44.67
**MxBR-93**	-46.67	23.00	-10.71	-9.09	-15.90	-4.54	-32.84	-16.78
**MxBR-94**	-54.33	16.10	77.39	80.62	-9.81	2.38	-29.37	-12.48
**MxCR-5**	-60.44	10.60	-59.79	-37.60	-7.43	5.08	-16.16	3.89
**MxCR-6**	-30.56	37.50	-43.56	-12.40	-3.24	9.84	-11.23	10.00
**MxCR-7**	-47.22	22.50	-86.45	-78.98	-10.19	1.95	-29.02	-12.04
**MxCR-8**	-49.78	20.20	-62.89	-42.40	7.52	22.05	-14.70	5.70
**FRxBR-1**	-39.67	11.20	-44.83	-42.86	-20.10	-10.83	-30.69	-15.29
**FRxBR-2**	-31.33	16.20	-34.48	-32.14	-27.03	-18.56	-35.95	-21.71
**FRxBR-3**	-25.67	19.60	24.14	28.57	-34.26	-26.63	-32.53	-17.53
**FRxBR-4**	-18.50	23.90	-39.07	-36.89	-31.39	-23.43	-40.04	-26.71
**FRxBR-5**	-43.17	9.10	-58.62	-57.14	-39.60	-32.60	-44.95	-32.71
**FRxBR-6**	-43.00	9.20	-35.62	-33.32	-36.93	-29.61	-38.13	-24.38
**FRxBR-7**	-38.67	11.80	-60.34	-58.93	-11.78	-1.55	-20.33	-2.62
**FRxBR-8**	-31.33	16.20	-13.79	-10.71	26.83	41.55	-6.00	14.89
**FRxBR-9**	-29.00	17.60	-43.10	-41.07	-1.88	9.50	-22.80	-5.64
**FRxBR-10**	-21.17	22.30	-70.69	-69.64	-29.31	-21.10	-44.65	-32.36
**FRxBR-11**	-42.83	9.30	-48.28	-46.43	-18.42	-8.95	-41.76	-28.82
**FRxBR-12**	-48.00	6.20	-56.90	-55.36	-1.98	9.39	-13.24	6.04
**FRxBR-13**	-19.83	23.10	-58.62	-57.14	-38.12	-30.94	-53.55	-43.22
**FRxBR-14**	-21.67	22.00	0.00	3.57	4.26	16.35	-16.65	1.87
**FRxBR-15**	-39.00	11.60	-47.14	-45.25	-37.33	-30.06	-33.84	-19.13
**FRxBR-16**	2.00	36.20	18.97	23.21	-37.23	-29.94	-39.15	-25.62
**FRxBR-17**	-40.00	11.00	-21.83	-19.04	-45.94	-39.67	-46.07	-34.09
**FRxBR-18**	-46.33	7.20	-3.45	0.00	-39.41	-32.38	-46.44	-34.53
**FRxBR-19**	-42.83	9.30	54.03	59.54	-3.96	7.18	-26.55	-10.22
**FRxBR-20**	3.83	37.30	-23.00	-20.25	-32.48	-24.64	-40.85	-27.71
**FRxBR-21**	-29.67	17.20	-25.86	-23.21	15.15	28.51	-23.78	-6.84
**FRxBR-23**	-39.67	11.20	1.72	5.36	-36.73	-29.39	-44.71	-32.42
**FRxBR-24**	-34.00	14.60	-33.34	-30.96	-3.76	7.40	-18.04	0.18
**FRxBR-25**	-31.67	16.00	-48.28	-46.43	-34.26	-26.63	-37.67	-23.82
**FRxBR-26**	-44.00	8.60	-27.59	-25.00	-25.25	-16.57	-43.16	-30.53
**FRxBR-27**	-26.33	19.20	-27.59	-25.00	-44.65	-38.23	-49.20	-37.91
**FRxBR-28**	-22.83	21.30	37.93	42.86	-33.56	-25.86	-48.44	-36.98
**FRxBR-29**	-21.33	22.20	-11.48	-8.32	1.49	13.26	-17.47	0.87
**FRxBR-30**	-31.67	16.00	43.10	48.21	-24.85	-16.13	-35.31	-20.93
**FRxBR-31**	-42.17	9.70	-27.59	-25.00	3.27	15.25	-19.22	-1.27
**FRxBR-32**	-35.50	13.70	-51.72	-50.00	-30.69	-22.65	-44.02	-31.58
**FRxBR-33**	-34.67	14.20	-65.52	-64.29	-37.82	-30.61	-60.85	-52.16
**FRxBR-34**	-2.33	33.60	31.03	35.71	-16.24	-6.52	-34.18	-19.56
**FRxBR-35**	-48.00	6.20	0.00	3.57	-27.43	-19.01	-38.75	-25.13

**FRxBR-36**	-43.00	9.20	12.07	16.07	-13.07	-2.98	-26.76	-10.49
**FRxBR-37**	-38.33	12.00	-31.03	-28.57	-42.97	-36.35	-56.76	-47.16
**FRxBR-38**	13.00	42.80	-13.79	-10.71	-19.90	-10.61	-42.16	-29.31
**FRxBR-39**	-29.00	17.60	-59.76	-58.32	-1.09	10.39	-26.07	-9.64
**FRxBR-40**	-23.83	20.70	-74.72	-73.82	-54.06	-48.73	-69.02	-62.13
**FRxBR-41**	-19.50	23.30	33.62	38.39	-37.82	-30.61	-45.91	-33.89
**FRxBR-42**	-45.83	7.50	-51.17	-49.43	-29.70	-21.55	-41.60	-28.62
**FRxBR-43**	-42.00	9.80	-52.66	-50.96	-38.61	-31.49	-45.18	-33.00
**FRxCR-2**	-23.67	20.80	-84.54	-76.19	-38.80	-34.44	-35.87	-29.46
**FRxCR-5**	27.00	51.20	-36.08	-1.59	-22.40	-16.87	-24.25	-16.68
**FRxCR-8**	-2.83	33.30	-53.30	-28.10	-47.21	-43.45	-49.89	-44.88
**FRxCR-9**	-41.00	10.40	-17.32	27.30	0.86	8.05	-5.85	3.56
**FRxCR-10**	-38.67	11.80	-80.41	-69.84	-16.48	-10.53	-23.00	-15.30
**FRxCR-11**	-35.17	13.90	60.37	146.92	-47.38	-43.63	-52.95	-48.24
**FRxCR-12**	-24.67	20.20	-81.44	-71.43	-48.58	-44.92	-46.27	-40.90
**FRxCR-15**	7.67	39.60	-80.41	-69.84	-17.68	-11.82	-24.22	-16.64

L: Layla, S: Samourai, A: Avalanche, SA: Sweet Avalanche, M: Magnum, FR: First Red, BR: Black rose, CR: Cabbage rose; Ht: heterosis, Hbt: heterobeltiosis.

Table S2. Descriptive statistics.

**Traits**	**N**	**Avg.**	**Medyan**	**Max**	**Min**	**Sd**	**Skewness**	**Kurtosis**
Flower stem length	258	40.36	39.20	87.20	6.30	11.96	0.30	2.09
Petal number	258	31.74	28.54	155.56	6.67	18.36	2.28	9.62
Flower diameter	258	7.52	7.41	12.81	3.78	1.77	0.41	-0.31
Flower bud length	258	3.41	3.37	5.97	1.48	0.80	0.24	-0.03
Log-petal number	258	3.32	3.35	5.05	1.90	0.52	0.02	0.19

N: number of observations, Avg.: average, Sd: standard deviation.

Table S3. Characteristics of F_1 progenies.

F_1 No	Flower Stem Length (cm)	Petal Number	Scent	Flower Diameter (cm)	Bud Length (cm)
**LxBR-1**	63,20	28,00	scentless and barely perceptible	6,90	3,54
**LxBR-2**	11,20	14,67	scentless and barely perceptible	5,30	2,15
**LxBR-3**	36,50	25,67	scentless and barely perceptible	9,66	4,33
**LxBR-4**	31,20	36,33	slight-scented	7,00	3,44
**LxBR-5**	45,30	26,67	scentless and barely perceptible	6,90	3,12
**LxBR-6**	44,30	18,50	scentless and barely perceptible	6,75	3,15
**LxBR-7**	42,20	14,40	scentless and barely perceptible	5,78	2,35
**LxBR-8**	8,30	34,00	scentless and barely perceptible	5,50	2,20
**LxBR-10**	34,60	32,00	scentless and barely perceptible	7,90	3,81
**LxBR-11**	33,90	37,00	scentless and barely perceptible	9,50	4,09
**LxBR-12**	9,50	23,40	scentless and barely perceptible	5,13	2,26
**LxBR-13**	13,30	43,00	scentless and barely perceptible	5,58	2,15
**LxBR-14**	35,50	16,00	scentless and barely perceptible	7,15	2,87
**LxBR-15**	33,20	27,83	scentless and barely perceptible	8,65	4,11
**LxBR-16**	38,70	18,00	scentless and barely perceptible	5,72	2,66
**LxBR-17**	39,40	34,00	scentless and barely perceptible	6,21	3,02
**LxBR-18**	48,90	23,10	scentless and barely perceptible	4,83	1,94
**LxBR-19**	47,90	24,67	scentless and barely perceptible	11,17	5,97
**LxBR-20**	46,80	36,00	scentless and	5,92	2,90

			barely perceptible		
**LxBR-22**	48,70	20,50	scentless and barely perceptible	7,05	3,34
**LxBR-23**	42,60	31,50	scentless and barely perceptible	5,76	2,67
**LxBR-24**	43,60	29,00	scentless and barely perceptible	7,28	3,47
**LxBR-25**	48,90	33,67	slight-scented	9,31	4,11
**LxBR-26**	35,10	37,50	scentless and barely perceptible	6,58	3,29
**LxBR-28**	10,20	31,50	scentless and barely perceptible	4,31	1,82
**LxBR-30**	17,40	12,67	scentless and barely perceptible	7,85	3,00
**LxBR-31**	35,10	13,54	scentless and barely perceptible	8,24	3,72
**LxBR-32**	36,10	24,67	scentless and barely perceptible	8,72	3,24
**LxBR-33**	38,20	15,00	scentless and barely perceptible	7,94	3,33
**LxBR-34**	23,50	28,67	scentless and barely perceptible	5,04	2,01
**LxBR-35**	27,40	36,00	scentless and barely perceptible	4,91	1,95
**LxBR-36**	58,90	23,30	scentless and barely perceptible	7,99	3,95
**LxBR-37**	37,60	23,50	slight-scented	7,63	3,36
**LxBR-38**	46,80	15,67	scentless and barely perceptible	6,95	3,13
**LxBR-41**	47,00	15,67	scentless and barely perceptible	5,87	2,21
**LxBR-42**	36,20	14,00	strong-scented	9,12	4,32
**LxBR-43**	30,90	28,80	scentless and barely perceptible	7,93	3,55
**LxBR-44**	47,10	35,00	scentless and barely perceptible	6,54	3,27
**LxBR-45**	44,40	47,50	scentless and barely perceptible	6,91	3,88
**LxBR-46**	43,20	61,67	scentless and	8,59	4,03

			barely perceptible		
**LxBR-48**	39,60	38,67	scentless and barely perceptible	5,87	2,54
**LxBR-49**	36,50	36,00	scentless and barely perceptible	6,20	3,83
**LxBR-50**	38,70	26,37	moderate-scented	10,30	5,07
**LxBR-51**	39,40	30,00	scentless and barely perceptible	8,71	3,69
**LxBR-52**	42,30	52,00	slight-scented	6,88	3,39
**LxBR-53**	18,60	31,33	scentless and barely perceptible	4,92	2,21
**LxBR-54**	39,50	45,00	scentless and barely perceptible	5,50	2,32
**LxBR-55**	37,80	19,25	scentless and barely perceptible	8,33	3,59
**LxBR-56**	36,60	38,33	scentless and barely perceptible	8,99	3,74
**LxBR-57**	21,40	109,33	scentless and barely perceptible	8,11	4,38
**LxBR-58**	38,80	29,33	moderate-scented	7,73	3,13
**LxBR-59**	24,50	71,00	scentless and barely perceptible	5,43	2,25
**LxBR-61**	38,20	52,67	scentless and barely perceptible	7,56	3,79
**LxBR-62**	35,60	39,00	scentless and barely perceptible	7,40	3,89
**LxBR-63**	45,10	49,33	scentless and barely perceptible	7,79	3,78
**LxBR-64**	37,50	29,67	slight-scented	7,38	4,15
**LxBR-65**	35,10	20,67	scentless and barely perceptible	7,60	3,58
**LxBR-66**	41,10	17,67	scentless and barely perceptible	7,77	3,15
**LxBR-67**	48,00	23,60	scentless and barely perceptible	9,27	4,02
**LxBR-68**	47,10	37,67	scentless and barely perceptible	8,14	3,55
**LxBR-70**	47,40	27,00	scentless and barely perceptible	5,96	2,68

**LxBR-71**	34,10	33,33	scentless and barely perceptible	5,30	2,43
**LxBR-72**	38,90	24,33	scentless and barely perceptible	4,88	2,60
**LxBR-73**	41,50	24,33	slight-scented	8,20	3,52
**LxBR-74**	31,20	57,00	scentless and barely perceptible	9,46	4,05
**LxBR-75**	9,50	17,00	scentless and barely perceptible	5,50	2,32
**LxBR-76**	34,50	47,00	scentless and barely perceptible	8,89	3,98
**LxBR-78**	33,40	39,83	scentless and barely perceptible	8,74	3,67
**LxBR-79**	37,80	25,50	scentless and barely perceptible	7,93	3,36
**LxBR-80**	40,10	49,33	scentless and barely perceptible	9,57	3,68
**LxBR-81**	40,90	53,33	scentless and barely perceptible	6,47	2,88
**LxBR-82**	39,40	29,33	slight-scented	6,93	2,96
**LxBR-83**	45,10	32,00	scentless and barely perceptible	7,15	2,84
**LxBR-84**	46,20	17,25	scentless and barely perceptible	5,26	2,33
**LxBR-85**	47,80	25,00	scentless and barely perceptible	6,54	2,62
**LxBR-86**	44,80	48,67	scentless and barely perceptible	3,78	3,30
**LxBR-87**	13,60	16,67	scentless and barely perceptible	4,14	1,48
**LxBR-88**	36,10	30,00	scentless and barely perceptible	5,58	2,75
**LxCR-1**	35,50	38,00	moderate-scented	8,01	3,78
**LxCR-2**	38,50	108,67	scentless and barely perceptible	9,73	4,12
**LxCR-3**	45,20	40,00	slight-scented	8,56	4,26
**LxCR-5**	68,10	38,00	scentless and barely perceptible	9,99	4,27
**LxCR-9**	79,00	33,00	scentless and barely	7,26	3,44

			perceptible		
**LxCR-10**	34,10	13,00	scentless and barely perceptible	7,47	3,22
**SxBR-1**	38,50	55,00	scentless and barely perceptible	6,54	3,33
**SxBR-2**	31,50	6,67	scentless and barely perceptible	7,46	3,51
**SxBR-4**	48,10	36,00	scentless and barely perceptible	8,10	3,65
**SxBR-5**	47,50	12,67	scentless and barely perceptible	5,64	2,97
**SxBR-6**	46,20	30,00	slight-scented	6,85	3,35
**AxBR-1**	44,20	31,83	scentless and barely perceptible	11,73	5,01
**AxBR-2**	43,10	62,33	slight-scented	10,47	4,30
**AxBR-3**	67,30	28,75	slight-scented	9,68	4,11
**AxBR-4**	19,40	19,00	scentless and barely perceptible	6,87	2,88
**AxBR-5**	32,60	15,00	scentless and barely perceptible	10,66	4,60
**AxBR-6**	59,30	58,00	slight-scented	6,63	3,15
**AxBR-7**	39,20	61,20	scentless and barely perceptible	6,02	2,95
**AxBR-8**	35,50	22,33	scentless and barely perceptible	7,60	3,71
**AxBR-9**	36,10	26,09	scentless and barely perceptible	11,14	5,23
**AxBR-11**	18,90	31,25	scentless and barely perceptible	5,05	2,21
**AxBR-12**	33,30	53,67	scentless and barely perceptible	9,08	4,30
**AxCR-4**	35,60	83,33	slight-scented	5,99	2,39
**AxCR-5**	25,60	32,25	scentless and barely perceptible	8,99	3,94
**AxCR-6**	35,20	25,50	slight-scented	11,61	5,36
**AxCR-9**	38,70	34,00	scentless and barely perceptible	7,86	3,03
**SAxBR-1**	35,90	25,50	scentless and barely perceptible	6,18	3,33
**SAxBR-2**	38,20	37,00	scentless and barely	6,10	2,89

			perceptible		
**SAxBR-3**	42,10	30,67	scentless and barely perceptible	8,05	3,64
**SAxBR-4**	45,30	35,33	moderate-scented	7,98	3,36
**SAxBR-5**	61,20	33,67	scentless and barely perceptible	7,03	3,41
**SAxBR-7**	47,90	10,60	slight-scented	8,12	3,54
**SAxBR-8**	45,30	68,00	slight-scented	7,78	3,20
**SAxBR-9**	63,70	24,60	scentless and barely perceptible	7,37	3,30
**SAxBR-10**	66,90	39,67	scentless and barely perceptible	11,79	5,40
**SAxBR-11**	12,00	25,00	scentless and barely perceptible	4,66	2,16
**SAxBR-12**	32,00	15,10	scentless and barely perceptible	11,63	5,12
**SAxBR-13**	47,50	16,17	scentless and barely perceptible	8,63	3,64
**SAxBR-14**	68,10	66,67	strong-scented	8,95	3,70
**MxBR-1**	37,50	25,67	scentless and barely perceptible	5,99	2,57
**MxBR-2**	31,20	15,00	scentless and barely perceptible	8,28	3,88
**MxBR-3**	39,40	28,00	scentless and barely perceptible	7,18	2,85
**MxBR-4**	41,10	40,20	scentless and barely perceptible	9,41	4,32
**MxBR-6**	47,90	36,25	slight-scented	7,49	3,91
**MxBR-7**	45,30	26,00	scentless and barely perceptible	5,88	2,18
**MxBR-8**	38,70	34,67	slight-scented	9,31	4,09
**MxBR-9**	31,20	48,67	scentless and barely perceptible	5,03	2,98
**MxBR-10**	34,60	36,00	scentless and barely perceptible	5,98	2,85
**MxBR-11**	55,60	7,11	slight-scented	6,43	3,37
**MxBR-12**	39,00	41,67	scentless and barely perceptible	5,76	2,04
**MxBR-13**	41,00	21,00	slight-scented	7,78	3,55
**MxBR-14**	37,50	24,80	scentless and barely perceptible	9,74	4,56

**MxBR-15**	45,50	34,00	scentless and barely perceptible	6,43	2,99
**MxBR-16**	45,10	17,80	scentless and barely perceptible	8,70	3,81
**MxBR-17**	48,10	28,40	scentless and barely perceptible	7,98	3,52
**MxBR-18**	87,20	34,67	slight-scented	8,81	3,95
**MxBR-19**	18,20	22,00	scentless and barely perceptible	6,83	3,09
**MxBR-20**	30,70	20,50	scentless and barely perceptible	6,21	3,03
**MxBR-21**	26,80	25,67	slight-scented	4,91	2,02
**MxBR-22**	22,30	43,00	scentless and barely perceptible	7,87	3,22
**MxBR-23**	35,20	58,00	scentless and barely perceptible	8,25	2,71
**MxBR-24**	38,40	30,33	slight-scented	5,37	2,42
**MxBR-25**	36,70	26,00	scentless and barely perceptible	5,88	2,55
**MxBR-26**	39,20	50,33	scentless and barely perceptible	6,94	2,97
**MxBR-27**	48,20	12,80	slight-scented	7,97	3,05
**MxBR-28**	46,20	18,00	scentless and barely perceptible	5,35	2,95
**MxBR-29**	44,80	50,75	slight-scented	5,94	2,53
**MxBR-30**	46,20	92,00	slight-scented	8,13	3,71
**MxBR-31**	66,50	29,00	scentless and barely perceptible	8,08	3,57
**MxBR-32**	39,20	11,00	slight-scented	5,99	2,75
**MxBR-33**	33,10	23,67	scentless and barely perceptible	7,58	3,49
**MxBR-34**	35,70	61,67	scentless and barely perceptible	10,47	4,87
**MxBR-35**	18,20	20,00	scentless and barely perceptible	6,69	2,63
**MxBR-36**	36,20	27,00	slight-scented	7,33	3,80
**MxBR-37**	40,00	33,00	scentless and barely perceptible	5,32	2,43
**MxBR-38**	36,50	13,00	scentless and barely perceptible	8,32	3,64
**MxBR-39**	45,10	33,20	slight-scented	8,42	4,55

**MxBR-40**	48,00	35,75	scentless and barely perceptible	7,44	2,74
**MxBR-41**	30,60	21,50	scentless and barely perceptible	9,47	4,43
**MxBR-43**	45,60	51,20	slight-scented	5,72	2,05
**MxBR-44**	60,90	64,00	scentless and barely perceptible	7,41	3,39
**MxBR-45**	39,60	26,00	moderate-scented	8,63	4,17
**MxBR-46**	37,50	65,60	moderate-scented	9,82	4,48
**MxBR-47**	65,20	41,00	scentless and barely perceptible	7,97	3,74
**MxBR-48**	36,80	48,67	scentless and barely perceptible	8,89	3,82
**MxBR-49**	42,50	20,00	slight-scented	7,57	3,65
**MxBR-50**	45,80	70,75	moderate-scented	8,69	3,21
**MxBR-51**	47,10	15,75	scentless and barely perceptible	7,04	3,10
**MxBR-52**	37,60	50,80	scentless and barely perceptible	6,13	2,98
**MxBR-53**	41,90	31,67	moderate-scented	9,07	4,14
**MxBR-54**	67,30	9,70	scentless and barely perceptible	5,77	3,36
**MxBR-56**	42,80	57,67	scentless and barely perceptible	9,67	4,17
**MxBR-57**	36,20	28,00	slight-scented	9,54	4,14
**MxBR-58**	39,50	28,75	scentless and barely perceptible	7,94	3,93
**MxBR-59**	40,10	28,00	slight-scented	5,74	2,99
**MxBR-60**	26,90	25,00	scentless and barely perceptible	5,38	2,41
**MxBR-61**	35,60	29,67	scentless and barely perceptible	5,79	3,03
**MxBR-63**	37,80	31,00	slight-scented	7,83	3,70
**MxBR-64**	25,30	8,00	scentless and barely perceptible	4,08	2,12
**MxBR-65**	34,60	27,00	scentless and barely perceptible	5,11	2,29
**MxBR-66**	44,20	42,00	scentless and barely perceptible	5,60	2,87
**MxBR-67**	40,80	20,60	scentless and barely perceptible	6,50	2,87

**MxBR-68**	39,00	22,67	scentless and barely perceptible	7,67	3,67
**MxBR-69**	41,20	61,66	scentless and barely perceptible	6,55	3,26
**MxBR-70**	45,70	11,75	scentless and barely perceptible	5,54	2,85
**MxBR-71**	42,60	15,33	slight-scented	7,22	3,65
**MxBR-72**	47,80	25,67	scentless and barely perceptible	7,39	3,51
**MxBR-73**	11,20	27,00	scentless and barely perceptible	4,59	1,92
**MxBR-74**	65,50	13,00	scentless and barely perceptible	8,53	3,72
**MxBR-75**	43,00	29,00	scentless and barely perceptible	5,22	3,15
**MxBR-77**	44,20	14,80	scentless and barely perceptible	9,75	4,62
**MxBR-78**	6,30	36,00	scentless and barely perceptible	5,34	2,13
**MxBR-79**	35,50	50,33	scentless and barely perceptible	5,17	2,04
**MxBR-80**	31,90	14,00	scentless and barely perceptible	11,10	4,83
**MxBR-81**	38,70	16,00	scentless and barely perceptible	7,87	3,58
**MxBR-82**	36,20	30,00	scentless and barely perceptible	10,32	4,09
**MxBR-83**	33,20	20,00	scentless and barely perceptible	10,74	5,34
**MxBR-84**	41,20	18,28	scentless and barely perceptible	8,56	3,50
**MxBR-85**	45,60	39,00	scentless and barely perceptible	5,09	3,13
**MxBR-87**	61,30	49,00	scentless and barely perceptible	8,35	4,10
**MxBR-88**	35,60	23,00	scentless and barely perceptible	7,60	3,28
**MxBR-89**	33,60	29,00	scentless and barely	7,05	3,29

**MxBR-90**	37,50	35,00	perceptible moderate-scented	8,12	3,95
**MxBR-91**	38,50	62,67	scentless and barely perceptible	5,77	2,55
**MxBR-93**	48,00	25,00	scentless and barely perceptible	8,83	3,83
**MxBR-94**	41,10	49,67	scentless and barely perceptible	9,47	4,03
**MxCR-5**	35,60	39,00	scentless and barely perceptible	9,72	4,78
**MxCR-6**	62,50	54,75	moderate-scented	10,16	5,06
**MxCR-7**	47,50	13,14	scentless and barely perceptible	9,43	4,05
**MxCR-8**	45,20	36,00	scentless and barely perceptible	11,29	4,86
**FRxBR-1**	36,20	16,00	scentless and barely perceptible	8,07	3,81
**FRxBR-2**	41,20	19,00	scentless and barely perceptible	7,37	3,52
**FRxBR-3**	44,60	36,00	scentless and barely perceptible	6,64	3,71
**FRxBR-4**	48,90	17,67	scentless and barely perceptible	6,93	3,30
**FRxBR-5**	34,10	12,00	scentless and barely perceptible	6,10	3,03
**FRxBR-6**	34,20	18,67	scentless and barely perceptible	6,37	3,40
**FRxBR-7**	36,80	11,50	scentless and barely perceptible	8,91	4,38
FRxBR-8	41,20	25,00	scentless and barely perceptible	12,81	5,17
**FRxBR-9**	42,60	16,50	scentless and barely perceptible	9,91	4,25
**FRxBR-10**	47,30	8,50	scentless and barely perceptible	7,14	3,04
**FRxBR-11**	34,30	15,00	scentless and barely perceptible	8,24	3,20
**FRxBR-12**	31,20	12,50	scentless and barely perceptible	9,90	4,77

**FRxBR-13**	48,10	12,00	scentless and barely perceptible	6,25	2,56
**FRxBR-14**	47,00	29,00	scentless and barely perceptible	10,53	4,58
**FRxBR-15**	36,60	15,33	scentless and barely perceptible	6,33	3,64
**FRxBR-16**	61,20	34,50	scentless and barely perceptible	6,34	3,35
**FRxBR-17**	36,00	22,67	scentless and barely perceptible	5,46	2,97
**FRxBR-18**	32,20	28,00	scentless and barely perceptible	6,12	2,95
**FRxBR-19**	34,30	44,67	scentless and barely perceptible	9,70	4,04
**FRxBR-20**	62,30	22,33	scentless and barely perceptible	6,82	3,25
**FRxBR-21**	42,20	21,50	scentless and barely perceptible	11,63	4,19
**FRxBR-23**	36,20	29,50	scentless and barely perceptible	6,39	3,04
**FRxBR-24**	39,60	19,33	scentless and barely perceptible	9,72	4,51
**FRxBR-25**	41,00	15,00	scentless and barely perceptible	6,64	3,43
**FRxBR-26**	33,60	21,00	scentless and barely perceptible	7,55	3,13
**FRxBR-27**	44,20	21,00	scentless and barely perceptible	5,59	2,79
**FRxBR-28**	46,30	40,00	scentless and barely perceptible	6,71	2,84
**FRxBR-29**	47,20	25,67	scentless and barely perceptible	10,25	4,54
**FRxBR-30**	41,00	41,50	slight-scented	7,59	3,56
**FRxBR-31**	34,70	21,00	scentless and barely perceptible	10,43	4,44
**FRxBR-32**	38,70	14,00	scentless and barely perceptible	7,00	3,08
**FRxBR-33**	39,20	10,00	scentless and barely	6,28	2,15

			perceptible		
**FRxBR-34**	58,60	38,00	scentless and barely perceptible	8,46	3,62
**FRxBR-35**	31,20	29,00	scentless and barely perceptible	7,33	3,37
**FRxBR-36**	34,20	32,50	scentless and barely perceptible	8,78	4,03
**FRxBR-37**	37,00	20,00	scentless and barely perceptible	5,76	2,38
**FRxBR-38**	67,80	25,00	moderate-scented	8,09	3,18
**FRxBR-39**	42,60	11,67	scentless and barely perceptible	9,99	4,07
**FRxBR-40**	45,70	7,33	scentless and barely perceptible	4,64	1,70
**FRxBR-41**	48,30	38,75	slight-scented	6,28	2,98
**FRxBR-42**	32,50	14,16	scentless and barely perceptible	7,10	3,21
**FRxBR-43**	34,80	13,73	scentless and barely perceptible	6,20	3,02
**FRxCR-2**	45,80	15,00	slight-scented	7,13	3,53
**FRxCR-5**	76,20	62,00	scentless and barely perceptible	9,04	4,17
**FRxCR-8**	58,30	45,30	moderate-scented	6,15	2,76
**FRxCR-9**	35,40	80,20	slight-scented	11,75	5,18
**FRxCR-10**	36,80	19,00	scentless and barely perceptible	9,73	4,24
**FRxCR-11**	38,90	155,56	scentless and barely perceptible	6,13	2,59
**FRxCR-12**	45,20	18,00	scentless and barely perceptible	5,99	2,96
**FRxCR-15**	64,60	19,00	scentless and barely perceptible	9,59	4,17

L: Layla, S: Samourai, A: Avalanche, SA: Sweet Avalanche, M: Magnum, FR: First Red, BR: Black rose, CR: Cabbage rose.

1.1. Supplementary Figures

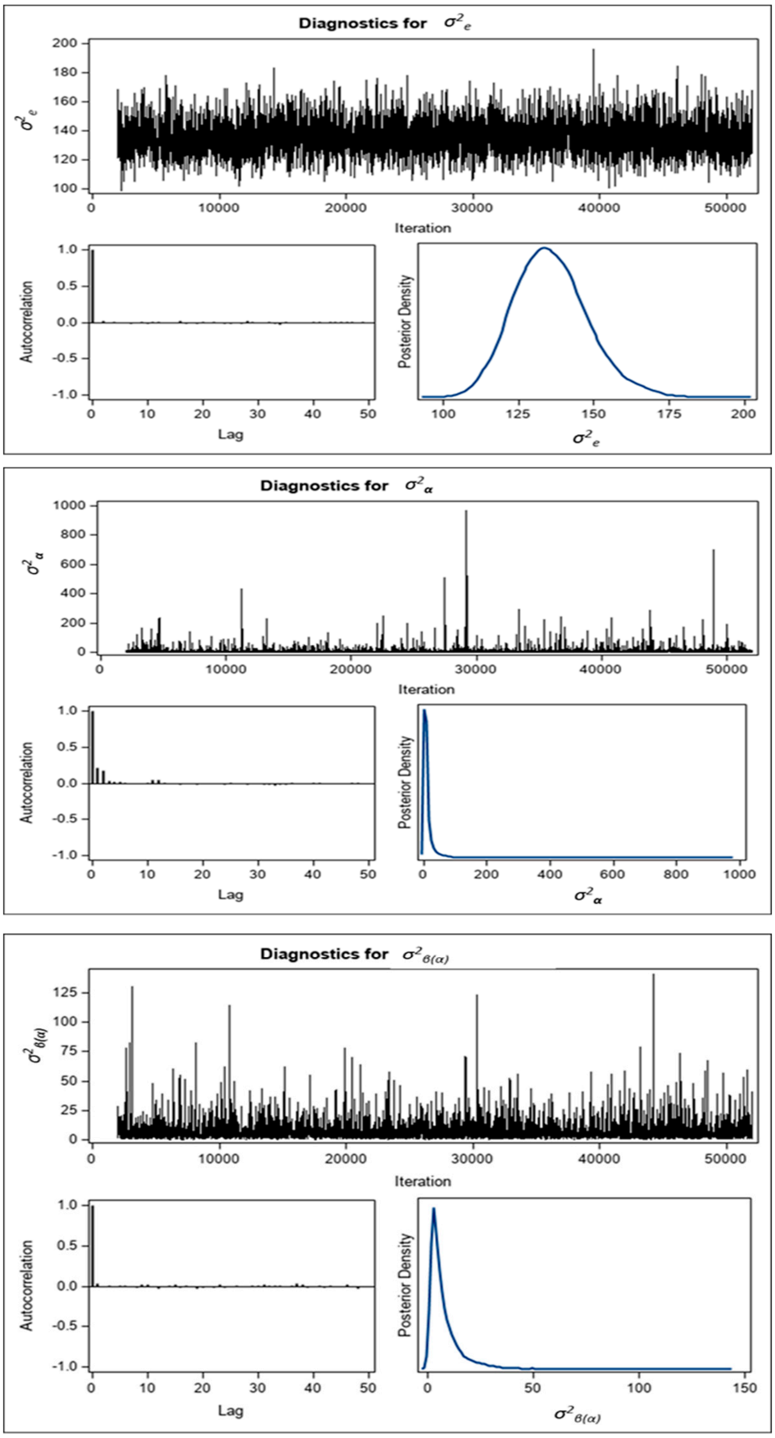

Figure S1. The posterior TAD (trace-autocorrelation density) panels for flower stem length of $\sigma^2{}_e$, $\sigma^2{}_\alpha$ and $\sigma^2{}_{\beta(\alpha)}$. The Markov chain converges very well with very low autocorrelation and almost a perfect normal posterior distribution in all TAD panels representing different parameters. Overall, this dataset is sufficient to allow more precise estimates of the parameters.

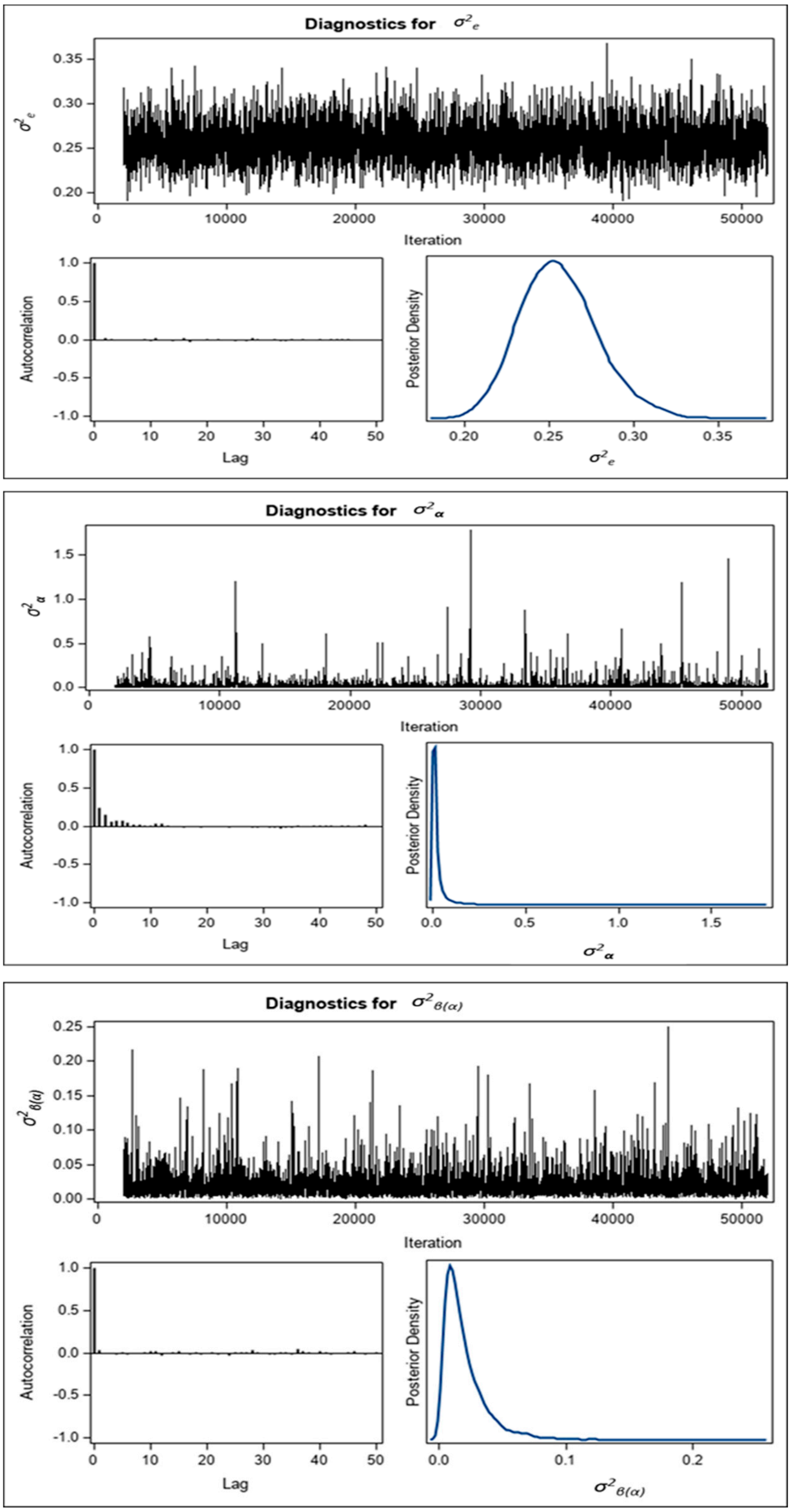

Figure S2. The posterior TAD (trace-autocorrelation density) panels for petal number of σ^2_e, σ^2_α and $\sigma^2_{\beta(\alpha)}$. The Markov chain converges very well with very low autocorrelation and almost a perfect normal posterior distribution in all TAD panels representing different parameters. Overall, this dataset is sufficient to allow more precise estimates of the parameters.

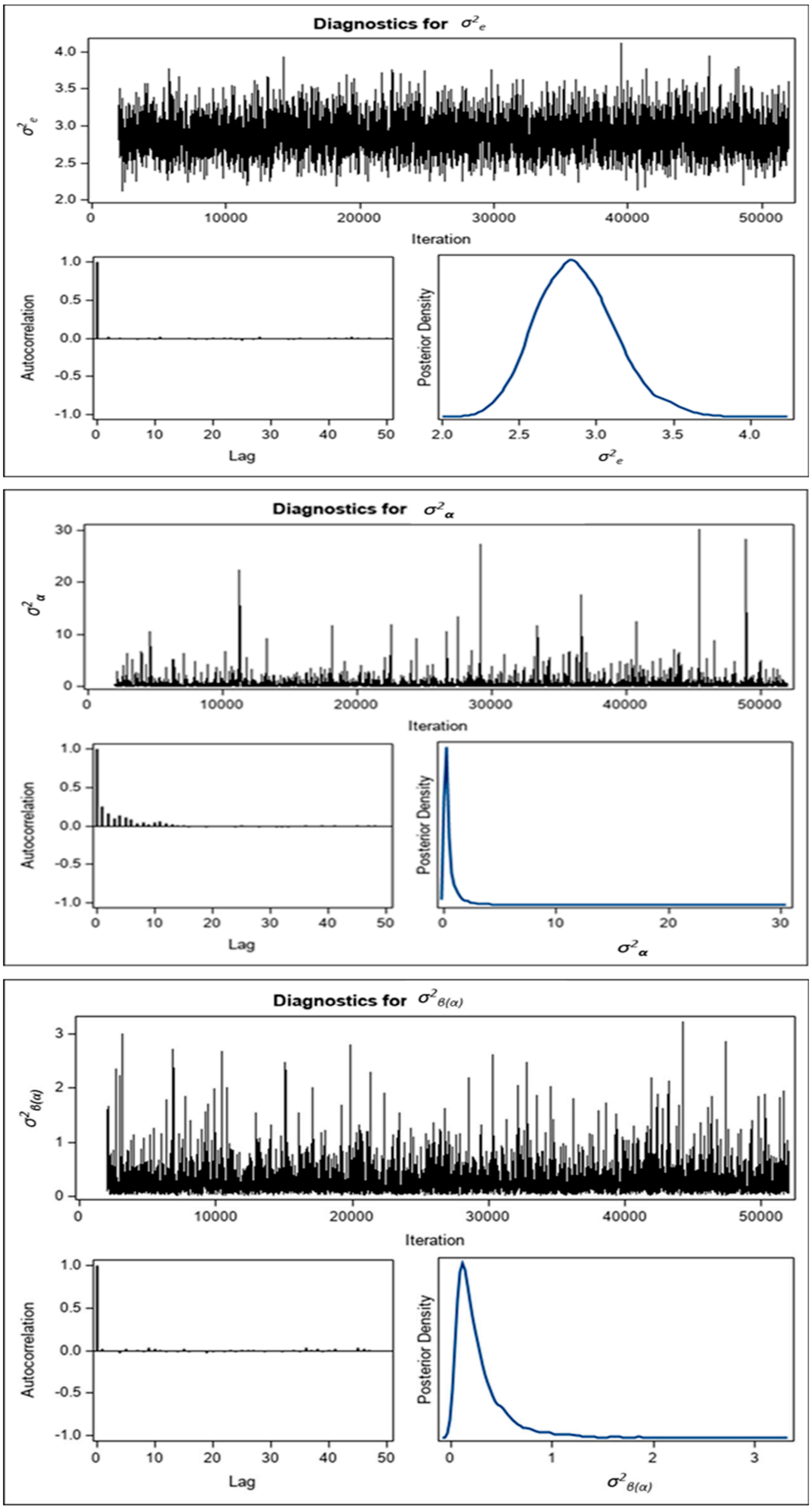

Figure S3. The posterior TAD (trace-autocorrelation density) panels for flower diameter of σ^2_e, σ^2_α and $\sigma^2_{\beta(\alpha)}$. The Markov chain converges very well with very low autocorrelation and almost a perfect normal posterior distribution in all TAD panels representing different parameters. Overall, this dataset is sufficient to allow more precise estimates of the parameters.

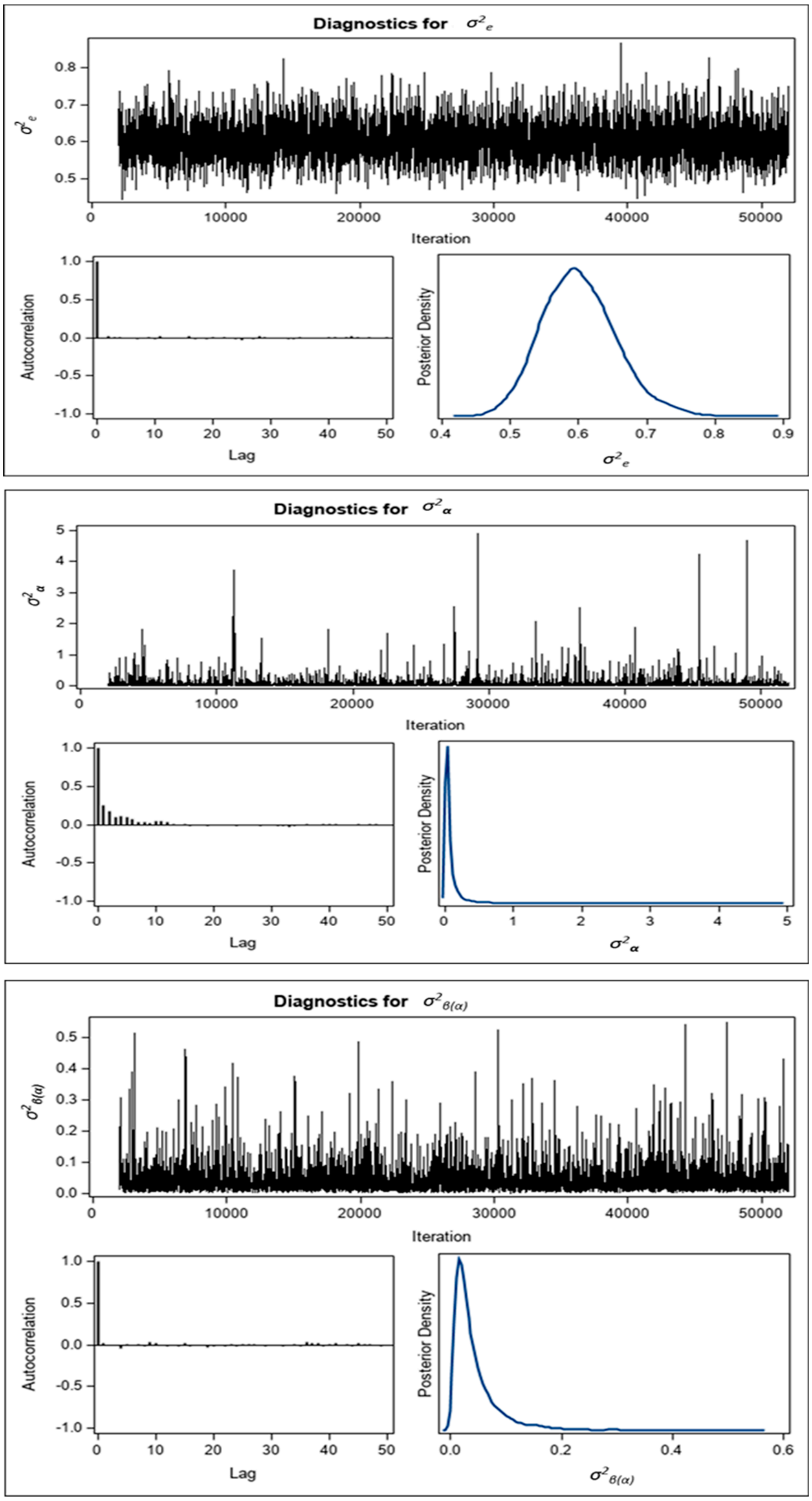

Figure S4. The posterior TAD (trace-autocorrelation density) panels for flower bud length of σ^2_e, σ^2_α and $\sigma^2_{\beta(\alpha)}$. The Markov chain converges very well with very low autocorrelation and almost a perfect normal posterior distribution in all TAD panels representing different parameters. Overall, this dataset is sufficient to allow more precise estimates of the parameters.

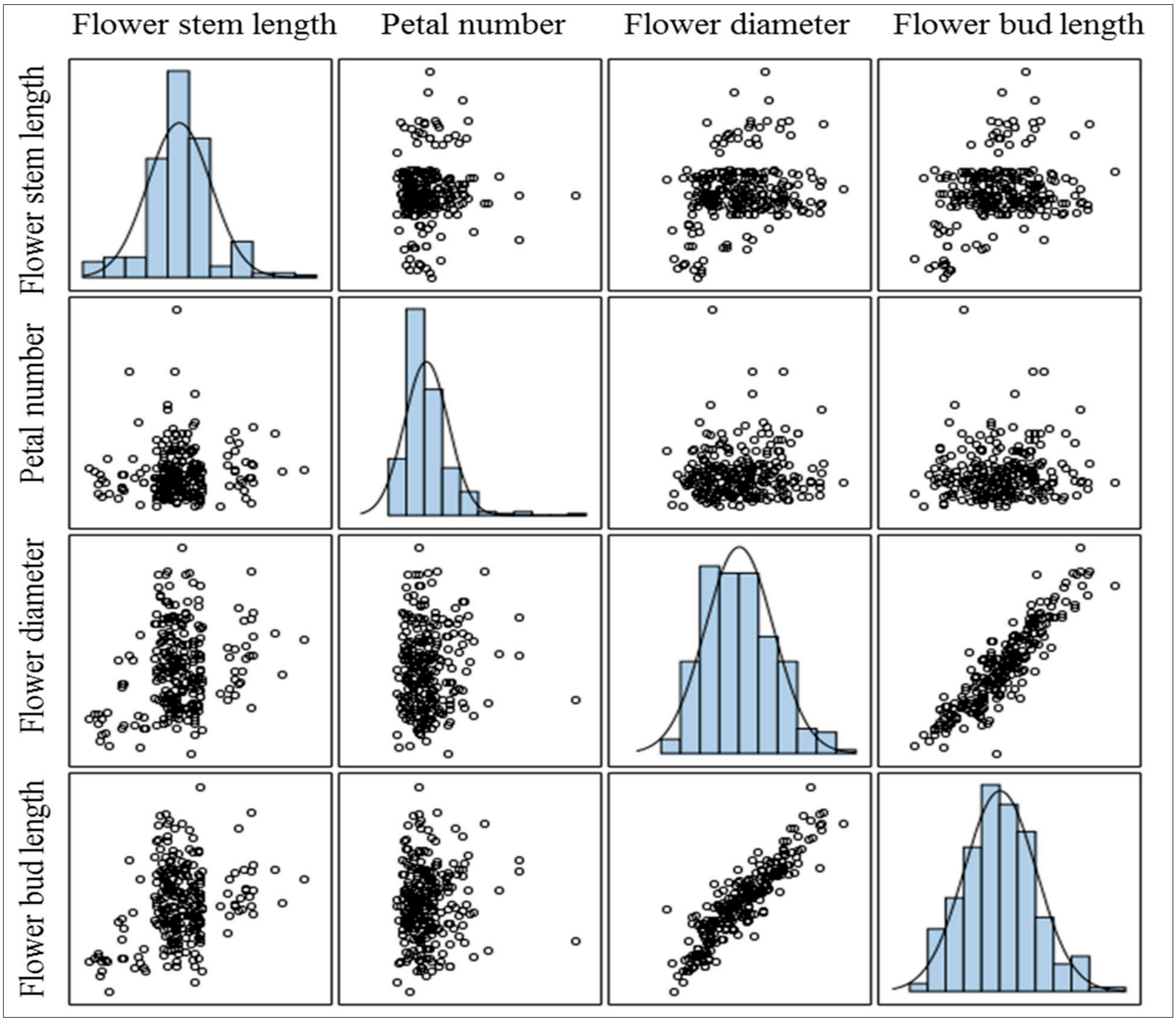

Figure S5. Phenotypic correlation matrix between quantitaive traits (p≤0.01).
